# Discovery of SNPs and InDels in papaya genotypes and its potential for marker assisted selection of fruit quality traits

**DOI:** 10.1038/s41598-020-79401-z

**Published:** 2021-01-11

**Authors:** Dieimes Bohry, Helaine Christine Cancela Ramos, Pedro Henrique Dias dos Santos, Marcela Santana Bastos Boechat, Fernanda Abreu Santana Arêdes, Adriana Azevedo Vimercati Pirovani, Messias Gonzaga Pereira

**Affiliations:** Universidade Estadual do Norte Fluminense Darcy Ribeiro - UENF, Campos dos Goytacazes, RJ CEP 28013-602 Brazil

**Keywords:** Next-generation sequencing, Genetic markers, Plant breeding

## Abstract

Papaya is a tropical and climacteric fruit that is recognized for its nutritional benefits and medicinal applications. Its fruits ripen quickly and show a drastic fruit softening, leading to great post-harvest losses. To overcome this scenario, breeding programs of papaya must invest in exploring the available genetic variation to continue developing superior cultivars with improved fruit quality traits. The objective of this study was to perform a whole-genome genotyping (WGG) of papaya, predict the effects of the identified variants, and develop a list of ripening-related genes (RRGs) with linked variants. The Formosa elite lines of papaya Sekati and JS-12 were submitted to WGG with an Illumina Miseq platform. The effects of variants were predicted using the snpEff program. A total of 28,451 SNPs having Ts/Tv (Transition/Transversion) ratio of 2.45 and 1,982 small insertions/deletions (InDels) were identified. Most variant effects were predicted in non-coding regions, with only 2,104 and 138 effects placed in exons and splice site regions, respectively. A total of 106 RRGs were found to be associated with 460 variants, which may be converted into PCR markers to facilitate genetic mapping and diversity studies and to apply marker-assisted selection (MAS) for specific traits in papaya breeding programs.

## Introduction

Papaya (*Carica papaya* L.) is a fruit crop cultivated in tropical and subtropical regions of the globe that is listed among the four major fresh tropical fruits. In Brazil, papaya is an important crop with a production of around 1.06 million tonnes in 2018, placing the country as the second major producer and the third major exporter, although with most of the production destined for the domestic market^1^. Papaya fruits are appreciated and highly indicated for their excellent nutritional and medicinal qualities, possessing high vitamin A and C content, antioxidants such as β-carotene and lycopene, minerals, and fibers^2,3^.

In papaya, several genetic and genomic resources are available due to the great advances of sequencing technologies, which have contributed to understand the intriguing sex-determination system of the species^4–8^. Besides the sex determination of papaya, other relevant traits have been investigated through gene expression analysis, such as the fruit quality-related traits^9–11^, embryogenesis^12^, resistance to drought^13^, etc. However, the utilization of sequencing technologies to identify DNA polymorphisms for the genetic mapping of important traits for papaya breeding is scarce. The available linkage maps for papaya have varied in coverage, resolution, and type of DNA polymorphisms. The first high-density linkage map was based on 1498 Amplified Fragment Length Polymorphisms (AFLP)^14^. The following high-density map was developed with 706 Simple Sequence Repeat (SSR) markers^15^. The same mapping population was used to improve the map resolution with 277 AFLP and 712 SSR markers and allowed the identification of 14 quantitative trait loci (QTL) related to fruit quality traits^16^. More recently, a linkage map based on 219 single nucleotide polymorphisms (SNP) was developed^17^. Although this map was based on SNP markers, the great distortion of the expected marker segregation observed in F_2_ (1:2:1) significantly decreased the map resolution. Still, a total of 21 QTLs for fruit quality traits were detected using this map and will enable candidate gene isolation and development of marker-assisted selection strategies.

DNA variants such as SNPs and InDels are very abundant in all genomes and are thought to bring out the phenotypic differences among individuals of a species, including differences related to yield and fruit quality traits^18–21^. SNPs and InDels are quickly identified through Next Generation Sequencing (NGS) technologies and numerous studies in climacteric fruit crops revealed the potential of NGS-based markers for the genetic mapping of fruit quality traits^17,22–24^.

Understanding the genetic and genomic aspects related to fruit quality traits in papaya is essential to continue developing superior cultivars with unique features to meet both the national and international markets. The conventional breeding of papaya for complex traits, such as fruit firmness and total soluble solids (TSS) content, is time-consuming and only gives small genetic gain per selection cycle. The ethylene is the main phytohormone regulating the ripening of climacteric fruits and its action influences the development of the sensorial and nutritional attributes of climacteric fruits^25^. One major change in texture during the ripening of such fruits is the rapid fruit softening, turning it more susceptible to physical injuries and post-harvest diseases. Fruit softening is a complex process with substantial activity of cell-wall degrading enzymes, such as polygalacturonase and beta-galactosidase^9,10^. Another problem of papaya breeding in Brazil is the occurrence of viral diseases that due to federal legislation the papaya plants even in breeding fields must be cut down when showing the first symptoms of viral diseases mainly the Papaya ringspot virus (PRSV), not allowing complete measurements in breeding populations. Thus, the use of molecular markers could speed up the time for selection in papaya breeding programs by allowing the analysis of a higher number of progenies at an early stage of development and increase the genetic gain^26^.

In Brazil, the papaya breeding program at UENF has had great success in the development of 21 new papaya cultivars^27^, which reduced the need to import hybrid seeds, expanded the options for farmers and consumers, and placed the Country as a potential papaya seed exporter. One of these cultivars is the UC10 hybrid, with fruits of around 1.9 kg and a high yield^28^. The parental of this hybrid are the Formosa elite lines Sekati and JS-12, which are contrasting for agronomic and fruit quality attributes. The Sekati parent produces large fruits, excellent pulp firmness, and median soluble solid contents. On the other hand, the JS12 parent diverges from Sekati in the last two traits, since it presents moderate pulp firmness and high soluble solid contents^29^. The availability of genomic information related to fruit quality traits will enable the development of tools to aid the selection process in papaya. Thus, in this study, we carried out a genome-wide identification of DNA variants among the Formosa elite lines Sekati and JS-12, using an Illumina MiSeq platform. The identified variants were used to predict its effects according to genomic location and to develop a list of ripening-related genes with linked variants to facilitate further genotype/phenotype association studies and to apply marker-assisted selection for the papaya breeding.

## Results

### SNP and InDel discovery and chromosomal distribution

A total of 12,709,090 sequence reads (with length ranging from 31 to 251 bp) were obtained from the Sekati and JS-12 lines. The Sekati sample generated 1.16 Gb of sequencing data (4,237,292 reads), while the JS-12 sample generated 2.4 Gb (8,471,798 reads). Mapping of the clean reads, after removing low quality reads, against the papaya reference genome resulted in the identification of 28,451 SNPs and 1,982 InDels (1,061 insertions and 921 deletions). The average coverage of variants was ~ 3.12× and ~ 5.02× for the Sekati and JS-12 lines, respectively.

**Figure 1:**
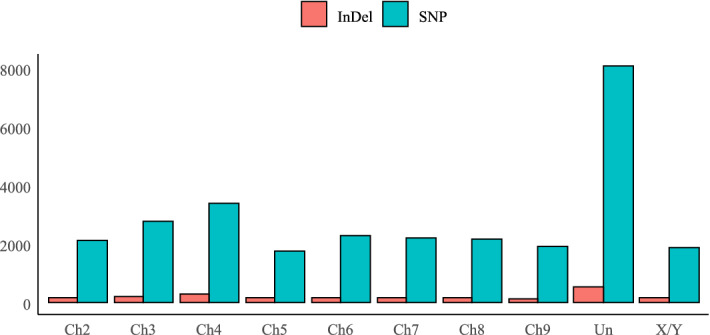
SNP and InDel distribution across the papaya chromosomes. *Ch* chromosome, *Un* unmapped contigs and scaffolds, *X/Y* sexual chromosomes XY.

The SNPs were identified in all nine papaya chromosomes (Fig. 1). The highest number of SNPs was observed on chromosome 4 (3,375 SNPs) and the lowest on chromosome 5 (1,751 SNPs). A total of 8,079 SNPs (28.4%) were identified in contigs and scaffolds that are not mapped to any papaya linkage group^15^ and they were attributed to unmapped contigs and scaffolds. The comparison of SNPs identified in the lines Sekati and the JS-12 revealed that they share about 78% (22,629) and 22% (5,822), respectively, of the genome-wide SNP alleles with the reference genome, which is the SunUp, a transgenic variety of the Solo heterotic group. The lines showed different levels of SNP similarities with the SunUp in all chromosomes. The Ch4 and Ch7 of Sekati shares about 94.3% and 82.1% of similarity with the reference genome, respectively. On Ch6, Ch9, and Ch8 the similarity of Sekati with the reference is the less, showing about 68.3%, 70.66%, and 71.5% of similarity, respectively. The remaining chromosomes of Sekati presented the similarity of SNPs close to the genome-wide average. The highest similarities of JS-12 alleles with the reference were observed on Ch6 (31.7%), Ch9 (29.34%), and Ch8 (28.5%). On Ch4 and Ch7 the similarity was 5.7% and 17.9%, respectively. The allele similarities for the remaining chromosomes of JS-12 were close to the genome-wide average.

The InDels were found in all nine papaya chromosomes (Fig. 1). The highest InDel number was found to be 260 on Ch4, while the lowest was 112 on Ch9. A total of 529 InDels were observed on unmapped contigs and scaffolds.

Based on nucleotide substitutions, the SNPs were classified as transitions (purine-purine and pyrimidine-pyrimidine) or transversions (purine-pyrimidine and pyrimidine-purine). We found 20,199 transitions and 8,252 transversions, with a genome-wide transition to transversion ratio (Ts/Tv) of 2.45. Observation of SNPs in coding regions revealed that the nucleotide substitution frequency and the Ts/Tv ratio were higher at the third codon position (2.40), compared to the second (1.96) and first (1.83) codon positions (Table 1).

**Table 1:** Frequency and type of nucleotide substitutions at codon sites.

Codon position	Transitions (Ts)	Transversions (Tv)	Ts/Tv ratio
3°	755	315	2.40
2°	350	179	1.96
1°	286	156	1.83
Total	1,391	650	2.14

### Functional classification of DNA variants

**Figure 2:**
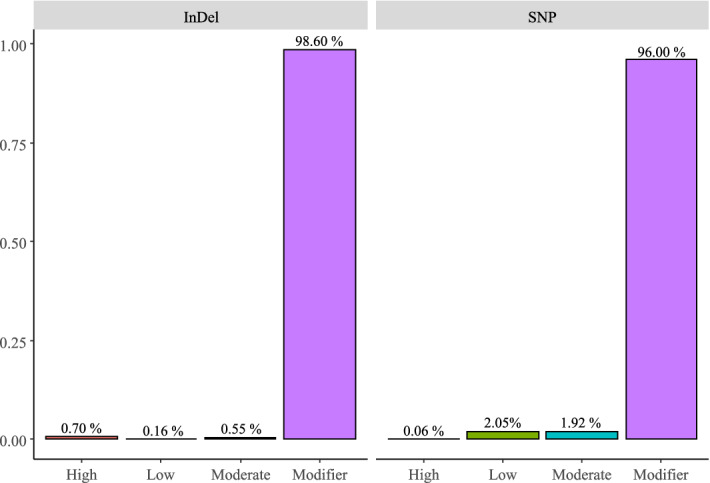
Number of InDel and SNP effects by impact.

A total of 58,498 effects based on genomic position were predicted from 30,433 DNA variants. The higher number of effects compared with the number of variants is because one specific variant can affect multiple genes (e.g. a variant can be downstream from one gene and upstream from another gene). The SNPs and InDels caused a total of 54,100 and 4,398 (Fig. 2) effects, respectively. The effects of the variants were classified into four categories: modifier (56,380), low (1,117), moderate (1,062), and high (63) impact. Only 4% and 1.4% of the SNP and InDel effects, respectively, were placed in coding regions.

**Figure 3:**
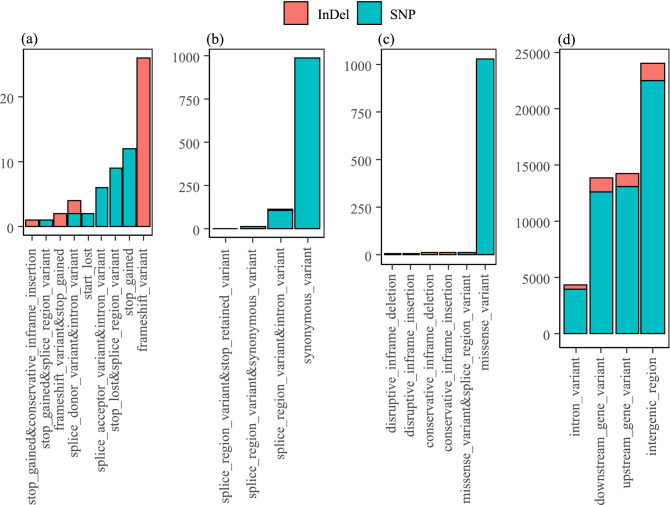
Distribution of SNP and InDel effects in the sub-classification of (**a**) high, (**b**) moderate, (**c**) low, and (**d**) modifier impacts.

High impact variants had a direct impact on gene functionality. A total of 32 and 31 high impact variants were observed for SNPs and InDels, respectively. The most common effects caused by high impact SNPs are stop codon lost and stop codon gain (Fig. 3a), which may lead to a high level of functional consequences. Meanwhile, high impact InDels mainly caused disruption of the translational reading frame and may result in abnormal protein products with an incorrect amino acid sequence. Moderate impact SNPs caused a change in one amino acid due to a non-synonymous substitution (Fig. 3b). The InDels caused four types of effects in coding regions that were classified as moderate impact (Fig. 3b). Low impact SNPs mainly consisted of synonymous substitutions in which no change of amino acid is observed (Fig. 3c). The remaining effects were predicted in non-coding regions and they were classified as modifier impact (Fig. 3d).

### Identification of fruit ripening-related genes with linked variants

We selected 48 differentially expressed genes (DEGs) during the fruit ripening process of papaya determined by RNAseq, including 20 cell wall-related genes (CW), 13 chlorophyll and carotenoid metabolism-related genes (CCM), four proteinases and their inhibitors (PROT), six plant hormone signal transduction pathway genes (PH), four transcription factors (TF), and one senescence-associated gene (SEN)^10^. These genes were used as Blastp queries to identify other ripening-related genes within the papaya genome. This search resulted in the identification of other 143 genes that are potentially involved in the fruit ripening process due to sequence similarity.

From 191 selected ripening-related genes (48 DEGs and 143 identified by BLASTp), a total of 106 genes were found to be associated with 460 variants (438 SNPs and 22 InDels) (Supplementary S1). The 106 ripening-related genes (36 DEGs and 70 identified by BLASTp) with linked variants were classified into five categories: cell wall-related genes (55), chlorophyll and carotenoid metabolism-related genes (10), proteinases and their inhibitors (13), plant hormone signal transduction pathway genes (11) and transcription factors (17). Most of these variants are located in the flanking regions of the RRGs, including 206 variants in intergenic regions that are no farther than 40 kb from the gene and 196 variants downstream/upstream of the genes. Only 58 variants are located inside the genes, including 36 in introns and 22 in exons. The exonic variants are separated as synonymous and missense variants (Table 2).

**Table 2:** Fruit ripening-related genes with low and moderate SNP impacts.

Gene ID	Description	Function	Gene type	Chr	Variant ID	Effect	AA change
evm.model.supercontig_43.43	Hydroxymethylbilane synthase	CCM	DEG	Ch6	SNP_43.43[A/G]569749	SV	Ser384Ser
evm.model.supercontig_92.51	Magnesium-chelatase subunit chlh	CCM	DEG	Ch8	SNP_92.51[G/A]470716	SV	Leu675Leu
evm.model.supercontig_43.43	Hydroxymethylbilane synthase	CCM	DEG	Ch6	SNP_43.43[G/C]569757	MV	Cys387Ser
evm.model.supercontig_151.20	Glycosyl hydrolase 9B13	CW	BLAST	Ch5	SNP_151.20[G/C]175639	SV	Ser435Ser
evm.model.supercontig_106.54	Glycosyl hydrolase family protein	CW	DEG	Ch6	SNP_106.54[C/T]643444	SV	Pro679Pro
					SNP_106.54[T/G]643459	SV	Val674Val
					SNP_106.54[T/G]643531	SV	Leu650Leu
evm.model.supercontig_29.125	Glycosyl hydrolases family 32 protein	CW	BLAST	Ch4	SNP_29.125[G/A]1173309	SV	Asp379Asp
evm.model.supercontig_508.5	Major facilitator superfamily protein	CW	BLAST	Ch3	SNP_508.5[C/T]19647	SV	Thr486Thr
evm.model.supercontig_25.194	Pectin methylesterase 3	CW	BLAST	Ch4	SNP_25.194[G/A]2039032	SV	Tyr61Tyr
					SNP_25.194[G/A]2039146	SV	Ile23Ile
evm.model.supercontig_82.65	Sucrose synthase 4	CW	DEG	Un	SNP_82.65[A/C]1098666	SV	Ala803Ala
evm.model.supercontig_93.34	Beta-galactosidase 12	CW	DEG	Ch7	SNP_93.34[T/C]879460	MV	Val624Ala
evm.TU.contig_32583.1	Beta-galactosidase 3	CW	BLAST	Un	SNP_32583.1[C/T]2456	MV	Gly233Ser
evm.model.supercontig_119.14	Expansin A1	CW	BLAST	Ch6	SNP_119.14[G/A]71404	MV	Arg225His
evm.model.supercontig_14.96	Expansin A5	CW	BLAST	X/Y	SNP_14.96[C/T]1096729	MV	Ser17Asn
evm.model.supercontig_3.313	Glycosyl hydrolases family 32 protein	CW	DEG	Ch9	SNP_3.313[G/C]2180909	MV	Glu637Gln
evm.model.supercontig_25.194	Pectin methylesterase 3	CW	BLAST	Ch4	SNP_25.194[G/A]2039051	MV	Ala55Val
evm.model.supercontig_151.32	Ethylene response sensor 1	PH	DEG	Ch5	SNP_151.32[A/T]288630	SV	Val331Val
evm.model.supercontig_6.74	Auxin-responsive GH3 family protein	PH	BLAST	Ch4	SNP_6.74[G/A]571741	MV	Ala6Val
evm.model.supercontig_152.36	WRKY family transcription factor	TF	BLAST	X/Y	SNP_152.36[C/T]269630	SV	Ser551Ser
evm.model.supercontig_9.36	WRKY DNA-binding protein 48	TF	BLAST	Ch5	SNP_9.36[A/T]151586	MV	Gln308His

*AA* amino acid, *MV* missense variant, *SV* synonymous variant, *CW* cell wall-related genes, *CCM* chlorophyll and carotenoid metabolism-related genes, *PH* plant hormone signal transduction pathway genes, *TF* transcription factor.

## Discussion

The frequency of SNPs and the Ts/Tv ratio was higher at the third codon position, compared with the second and first codon position (Table 1), revealing a trend of genomic conservation at codon sites during evolution. This trend was also observed in SNPs identified in Expressed Sequence Tags (ESTs) from *Solanum lycopersicum* and *S. habrochiates*^27^.

SNPs are known to be associated with many quantitative trait loci in plants^20,30–33^ and an individual SNP can have a large impact on the phenotype^34,35^. We found 2180 SNPs located in coding regions and 26,271 in non-coding regions of the papaya genome. Although most SNPs are not located inside genes, their abundance and robustness make them an important source of DNA variation to help papaya breeding programs in the development of superior cultivars. InDels also play important roles in the phenotypic variation observed between individuals of a species. In papaya, a dinucleotide insertion mutation in the gene encoding the enzyme lycopene β-cyclase (CpCYC-b) causes the phenotypic variation of red and yellow flesh^36^. When found in coding regions the InDels generally disrupt the translational reading frame (frameshift variant), except when the mutation is a multiple of three nucleotides^37^. In this study, we identified 62 InDels located in coding regions and 28 of these causing disruptions of the translational reading frame.

Fruit quality is one of the most important features pursued by papaya breeding programs, especially the selection of genotypes that keep fruit firmness for a longer period, resulting in longer shelf-life and decrease post-harvest losses. Studies at the gene expression level were developed to isolate the key genes underlying the fruit ripening process and fruit softening of papaya^9,10,38^. However, these studies analyzed only one genotype at a time and not considered the variation within DNA sequences among different papaya genotypes.

During the ripening process of climacteric fruits such as papaya and peach, a positive feedback loop regulated by NAC transcription factor is thought to control the ethylene synthesis. This mechanism is observed in species that lack recent whole-genome duplication (WGD). On the other hand, climacteric fruit species with recent WGD, such as tomato, pear, and apple, appear to have evolved a MADS-type transcription factor positive feedback loop controlling ripening^25^. Fruit softening in papaya is mainly caused by the degradation of primary cell wall polymers. Several cell wall-degrading enzymes act cooperatively in a coordinated process to degrade the cellulose-hemicellulose matrix which is embedded in a structurally heterogeneous mixture of pectin^39^. While ethylene promotes fast fruit softening, on another hand it is also thought to improve the rate of sugar synthesis, transport, and degradation during the ripening of papaya. Several genes related to sugar metabolism are up-regulated in response to ethylene during the ripening process^10^. Plant hormones also play important roles in controlling several processes of growth and development in plants. Besides the importance of ethylene for the fruit ripening process, other types of plant hormones can take place synergically or antagonistically with the ethylene action during the ripening of climacteric fruits. Besides, one of the major physiological changes observed during the ripening of papaya is a fast color change^10,38^. This is because of the fast degradation of chlorophyll and the appearance of carotenoids such as lutein and β-carotene^11^. Other genes that are involved in fruit softening include the class of protease enzymes. Studies have shown that some proteases have increased expression during the ripening process of papaya^10^ and tomatoes^40^.

The availability of SNPs and InDels strongly associated with ripening-related genes of papaya is essential to develop studies of diversity, genetic mapping, and application of marker-assisted selection. Thus, we searched for DNA variants that are linked with ripening-related genes that are up or down-regulated in response to exogenous ethylene^10^ and genes identified using BLASTp. A total of 106 genes with at least one variant associated, either inside or in the flanking region of the gene, were identified (Supplementary S1). The association between an SNP and InDel with a trait of interest can be accessed through the linkage disequilibrium analysis^41^, using the quantitative trait (QTL) analysis for example. Further analysis will examine the genotype–phenotype association related to fruit ripening traits in a segregant population derived from the cross between the Sekati and JS-12 lines. It is expected that the presence of alleles for these fruit ripening-related genes in papaya germplasm and breeding populations can contribute to observed differences for the fruit firmness and TSS content among papaya genotypes. The association of genotypic alleles with a trait of interest points to a genomic region where one or more genes may be affecting the phenotype. To effectively apply MAS in breeding programs the candidate genes have to be identified and validated through functional analysis. After all these identification and validation steps, DNA markers based on PCR, such as the low-cost technique called single nucleotide amplified polymorphism (SNAP)^42^ or the real-time fluorescence-tagged probes technologies such as TaqMan, Kompetitive allele specific PCR (KASP), or rhAmp^43^, will be developed to apply marker-assisted selection and to direct gene editing studies in papaya breeding programs.

## Material and methods

### Plant materials

The Formosa elite lines of papaya Sekati and JS-12 were obtained from the UENF/CALIMAN germplasm bank and were cultivated in commercial fields at the Caliman Agrícola S.A. in Linhares-ES, Brazil. The Sekati line (originally from Malaysia) produces large fruits with excellent fruit firmness and moderate total soluble solid content. The JS-12 line (originally from the Embrapa—National Cassava & Fruits Research Center (CNPMF) of Brazil), on the other hand, presents high total soluble solid content and moderate fruit size and firmness^29,44^.

### Sequencing and variant identification

The genomic DNA was extracted from young leaves taken from one individual of each line using a Plant Genomics DNA Extraction Kit YGP 100—RBC (BioAmerica), following the manufacture instructions. The DNA concentration and quality were checked using a NanoDrop 2000 spectrophotometer (Thermo Scientific) and superfine resolution agarose gel (1%). The whole-genome libraries of the two lines were constructed using a Nextera library preparation kit (Illumina, Inc.), according to the manufacture instructions. Paired-ends (35–251 bp) fragments of the samples were sequenced with a MiSeq platform at the Laboratory of Biotechnology—LBT of the Universidade Estadual do Norte Fluminense. After sequencing, the quality of reads was checked using FastQC^45^. The filtered reads were aligned with the reference genome^46^ using Bowtie2^47^ with default parameters. Discovery and filtration of variants were carried out using SAMtools v0.1.18^48^. To facilitate visualizing the overall distribution of variants across the papaya chromosomes, the contigs and scaffolds of the reference genome, which is still a draft version, were associated with 10 papaya linkage groups (LGs)^15^ and the LGs with a pachytene chromosome-based karyotype of papaya^49^.

### Annotation of single nucleotide polymorphisms and insertion/deletion polymorphism

To predict the putative effects of DNA variants according to genomic location, the snpEff v4.3 program was used^37^. To perform the analysis a *C. papaya* binary database file (.bin) was built in snpEff using the papaya reference genome in Fasta format^46^ and an annotation file in gff3 format, both downloaded from the PLAZA: Comparative Genomics In Plants. A variant call format (VCF) file containing the SNPs and InDels was then annotated with the snpEff program using default parameters. The variants were classified as genic and intergenic according to their genomic location. The variants in intergenic regions are classified as Modifier impact and do not affect the coding regions of genes. Variants located in introns are classified as Modifier impact as well. The variants placed in coding genic regions can generate three types of impacts, such as low, moderate, and high impact. Low impact variants (e.g. synonymous variant) are assumed to be mostly harmless or unlikely to change protein behavior, while a non-disruptive variant that might change protein effectiveness is considered of moderate impact (e.g. missense variant and inframe deletion). The variants with high impact (e.g. stop gained and frameshift variant) probably cause protein truncation or loss of function^37^.

### Identification of fruit ripening-related genes with linked variants

To identify ripening-related genes, we selected 48 genes isolated from a differential gene expression experiment during the fruit ripening process of papaya fruits^10^. The protein sequences of the 48 differentially expressed genes (DEGs) were used as queries to identify genes with related function based on sequence similarity within the papaya genome. The Blastp tool available at Phytozome was used and the ripening-related genes were selected with a minimum of 50% identity and E-value ≤ 1e−20. We removed from the list of the ripening-related genes those identified by Blastp with no expression during fruit development and ripening of papaya^25^ and the genes without variants. We also removed the variants farther than 40 kb from the gene start/end.

## Supplementary Information

Supplementary Information.

## References

[1] FAOSTAT. *Food and Agriculture Organization of the United Nations* (2020). http://www.fao.org/faostat/en/#data/QC/visualize. Accessed 13 April 2020.

[2] Chandrika UG, Jansz ER, Wickramasinghe SMDN, Warnasuriya ND (2003). Carotenoids in yellow- and red-fleshed papaya (*Carica papaya* L.). J. Sci. Food Agric..

[3] De Souza LM, Ferreira KS, Chaves JBP, Teixeira SL (2008). L-ascorbic acid, B-carotene and lycopene content in papaya fruits (*Carica papaya*) with or without physiological skin freckles. Sci. Agric..

[4] Lee CY (2018). The development of functional mapping by three sex-related loci on the third whorl of different sex types of *Carica papaya* L.. PLoS ONE.

[5] Liao Z, Yu Q, Ming R (2017). Development of male-specific markers and identification of sex reversal mutants in papaya. Euphytica.

[6] VanBuren R (2015). Origin and domestication of papaya Y^h^ chromosome. Genome Res..

[7] VanBuren R (2016). Extremely low nucleotide diversity in the X-linked region of papaya caused by a strong selective sweep. Genome Biol..

[8] Ming R, Yu Q, Moore PH (2007). Sex determination in papaya. Semin. Cell Dev. Biol..

[9] Fabi JP (2014). Analysis of papaya cell wall-related genes during fruit ripening indicates a central role of polygalacturonases during pulp softening. PLoS ONE.

[10] Shen YH (2017). Isolation of ripening-related genes from ethylene/1-MCP treated papaya through RNA-seq. BMC Genomics.

[11] Shen YH (2019). Exploring the differential mechanisms of carotenoid biosynthesis in the yellow peel and red flesh of papaya. BMC Genomics.

[12] Jamaluddin ND, Mohd Noor N, Goh HH (2017). Genome-wide transcriptome profiling of *Carica papaya* L. embryogenic callus. Physiol. Mol. Biol. Plants.

[13] Gamboa-Tuz SD (2018). Transcriptomics and co-expression networks reveal tissue-specific responses and regulatory hubs under mild and severe drought in papaya (*Carica papaya* L.). Sci. Rep..

[14] Ma H (2004). High-density linkage mapping revealed suppression of recombination at the sex determination locus in papaya. Genetics.

[15] Chen C (2007). Construction of a sequence-tagged high-density genetic map of papaya for comparative structural and evolutionary genomics in Brassicales. Genetics.

[16] Blas AL (2012). Genetic mapping of quantitative trait loci controlling fruit size and shape in papaya. Mol. Breed..

[17] Nantawan U, Kanchana-udomkan C, Bar I, Ford R (2019). Linkage mapping and quantitative trait loci analysis of sweetness and other fruit quality traits in papaya. BMC Plant Biol..

[18] Huq A (2016). Identification of functional SNPs in genes and their effects on plant phenotypes. J. Plant Biotechnol..

[19] Larsen B (2019). Genome-wide association studies in apple reveal loci for aroma volatiles, sugar composition, and harvest date. Plant Genome..

[20] Nuñez-Lillo G (2019). High-density genetic map and QTL analysis of soluble solid content, maturity date, and mealiness in peach using genotyping by sequencing. Sci. Hortic. (Amsterdam).

[21] Liu X, Geng X, Zhang H, Shen H, Yang W (2017). Association and genetic identification of loci for four fruit traits in tomato using InDel markers. Front. Plant Sci..

[22] Luo C (2016). Construction of a high-density genetic map based on large-scale marker development in mango using specific-locus amplified fragment sequencing (SLAF-seq). Front. Plant Sci..

[23] Martínez-García PJ (2013). High density SNP mapping and QTL analysis for fruit quality characteristics in peach (*Prunus persica* L.). Tree Genet. Genomes.

[24] Sun R (2015). A dense SNP genetic map constructed using restriction site-associated DNA sequencing enables detection of QTLs controlling apple fruit quality. BMC Genomics.

[25] Lü P (2018). Genome encode analyses reveal the basis of convergent evolution of fleshy fruit ripening. Nat. Plants.

[26] Xu Y, Crouch JH (2008). Marker-assisted selection in plant breeding: From publications to practice. Crop Sci..

[27] MAPA. *Ministério da Agricultura, Pecuária e Abastecimento—Registro Nacional de Cultivares (RNC)* (2020). http://sistemas.agricultura.gov.br/snpc/cultivarweb/cultivares_registradas.php. Accessed 18 February 2020.

[28] Pereira MG (2019). UC10: A new early Formosa papaya cultivar. Crop Breed. Appl. Biotechnol..

[29] Cardoso DL, Nunes L, Maria C, De Macêdo P (2014). Heterosis in papaya: Inter and intragroup analysis. Rev. Bras. de Fruticult..

[30] Argyris JM (2017). QTL analyses in multiple populations employed for the fine mapping and identification of candidate genes at a locus affecting sugar accumulation in melon (*Cucumis melo* L.). Front. Plant Sci..

[31] Montero-Pau J (2017). An SNP-based saturated genetic map and QTL analysis of fruit-related traits in Zucchini using genotyping-by-sequencing. BMC Genomics.

[32] Celik I, Gurbuz N, Uncu AT, Frary A, Doganlar S (2017). Genome-wide SNP discovery and QTL mapping for fruit quality traits in inbred backcross lines (IBLs) of *solanum pimpinellifolium* using genotyping by sequencing. BMC Genomics.

[33] Pootakham W (2015). Genome-wide SNP discovery and identification of QTL associated with agronomic traits in oil palm using genotyping-by-sequencing (GBS). Genomics.

[34] Schreiber L, Nader-nieto AC, Schönhals EM, Walkemeier B (2014). SNPs in genes functional in starch-sugar interconversion associate with natural variation of tuber starch and sugar content of potato (*Solanum tuberosum* L.). G3 (Bethesda).

[35] Tzuri G (2015). A ‘golden’ SNP in CmOr governs the fruit flesh color of melon (*Cucumis melo*). Plant J..

[36] Blas AL (2010). Cloning of the papaya chromoplast-specific lycopene β-cyclase, CpCYC-b, controlling fruit flesh color reveals conserved microsynteny and a recombination hot spot. Plant Physiol..

[37] Cingolani P (2012). A program for annotating and predicting the effects of single nucleotide polymorphisms, SnpEff: SNPs in the genome of *Drosophila melanogaster* strain w1118; iso-2; iso-3. Fly (Austin).

[38] Fabi JP (2012). Analysis of ripening-related gene expression in papaya using an Arabidopsis-based microarray. BMC Plant Biol..

[39] Gapper NE, McQuinn RP, Giovannoni JJ (2013). Molecular and genetic regulation of fruit ripening. Plant Mol. Biol..

[40] Wang W, Cai J, Wang P, Tian S, Qin G (2017). Post-transcriptional regulation of fruit ripening and disease resistance in tomato by the vacuolar protease SlVPE3. Genome Biol..

[41] Flint-Garcia SA, Thornsberry JM, Buckler ES (2003). Structure of linkage disequilibrium in plants. Annu. Rev. Plant Biol..

[42] Drenkard E (2000). A simple procedure for the analysis of single nucleotide polymorphisms facilitates map-based cloning in Arabidopsis 1. Biochemistry.

[43] Broccanello C (2018). Comparison of three PCR-based assays for SNP genotyping in plants. Plant Methods.

[44] Cortes DFM (2019). Development of superior lines of papaya from the Formosa group using the pedigree method and REML/Blup procedure. Bragantia.

[45] Andrews, S. *FastQc—A Quality Control Tool for High Throughput Sequence Data* (2010). https://www.bioinformatics.babraham.ac.uk/projects/fastqc/. Accessed 4 April 2019.

[46] Ming R (2008). The draft genome of the transgenic tropical fruit tree papaya (*Carica papaya* L.). Nature.

[47] Langmead B, Salzberg SL (2012). Fast gapped-read alignment with Bowtie 2. Nat. Methods.

[48] Li H (2009). The sequence alignment/map format and SAMtools. Bioinformatics.

[49] Zhang W, Wai CM, Ming R, Yu Q, Jiang J (2010). Integration of genetic and cytological maps and development of a pachytene chromosome-based karyotype in papaya. Trop. Plant Biol..

